# Nanotechnology: A Revolution in Modern Industry

**DOI:** 10.3390/molecules28020661

**Published:** 2023-01-09

**Authors:** Shiza Malik, Khalid Muhammad, Yasir Waheed

**Affiliations:** 1 Bridging Health Foundation, Rawalpindi 46000, Pakistan; 2 Department of Biology, College of Science, UAE University, Al Ain 15551, United Arab Emirates; 3 Office of Research, Innovation, and Commercialization (ORIC), Shaheed Zulfiqar Ali Bhutto Medical University (SZABMU), Islamabad 44000, Pakistan; 4 Gilbert and Rose-Marie Chagoury School of Medicine, Lebanese American University, Byblos 1401, Lebanon

**Keywords:** nanotechnology, nanoindustries, agriculture, foods, medicine, textile, biotechnology, construction, environment, automobiles, cosmetics industry

## Abstract

Nanotechnology, contrary to its name, has massively revolutionized industries around the world. This paper predominantly deals with data regarding the applications of nanotechnology in the modernization of several industries. A comprehensive research strategy is adopted to incorporate the latest data driven from major science platforms. Resultantly, a broad-spectrum overview is presented which comprises the diverse applications of nanotechnology in modern industries. This study reveals that nanotechnology is not limited to research labs or small-scale manufacturing units of nanomedicine, but instead has taken a major share in different industries. Companies around the world are now trying to make their innovations more efficient in terms of structuring, working, and designing outlook and productivity by taking advantage of nanotechnology. From small-scale manufacturing and processing units such as those in agriculture, food, and medicine industries to larger-scale production units such as those operating in industries of automobiles, civil engineering, and environmental management, nanotechnology has manifested the modernization of almost every industrial domain on a global scale. With pronounced cooperation among researchers, industrialists, scientists, technologists, environmentalists, and educationists, the more sustainable development of nano-based industries can be predicted in the future.

## 1. Introduction

Nanotechnology has slowly yet deeply taken over different industries worldwide. This rapid pace of technological revolution can especially be seen in the developed world, where nano-scale markets have taken over rapidly in the past decade. Nanotechnology is not a new concept since it has now become a general-purpose technology. Four generations of nanomaterials have emerged on the surface and are used in interdisciplinary scientific fields; these are active and passive nanoassemblies, general nanosystems, and small-scale molecular nanosystems [1]. 

This rapid development of nanoscience is proof that, soon, nano-scale manufacturing will be incorporated into almost every domain of science and technology. This review article will cover the recent advanced applications of nanotechnology in different industries, mainly agriculture, food, cosmetics, medicine, healthcare, automotive, oil and gas industries, chemical, and mechanical industries [2,3]. Moreover, a brief glimpse of the drawbacks of nanotechnology will be highlighted for each industry to help the scientific community become aware of the ills and benefits of nanotechnology side by side. Nanotechnology is a process that combines the basic attributes of biological, physical, and chemical sciences. These processes occur at the minute scale of nanometers. Physically, the size is reduced; chemically, new bonds and chemical properties are governed; and biological actions are produced at the nano scale, such as drug bonding and delivery at particular sites [4,5].

Nanotechnology provides a link between classical and quantum mechanics in a gray area called a mesoscopic system. This mesoscopic system is being used to manufacture nanoassemblies of nature such as agricultural products, nanomedicine, and nanotools for treatment and diagnostic purposes in the medical industry [6]. Diseases that were previously untreatable are now being curtailed via nano-based medications and diagnostic kits. This technology has greatly affected bulk industrial manufacturing and production as well. Instead of manufacturing materials by cutting down on massive amounts of material, nanotechnology uses the reverse engineering principle, which operates in nature. It allows the manufacturing of products at the nano scale, such as atoms, and then develops products to work at a deeper scale [7]. 

Worldwide, millions and billions of dollars and euros are being spent in nanotechnology to utilize the great potential of this new science, especially in the developed world in Europe, China, and America [8]. However, developing nations are still lagging behind as they are not even able to meet the industrial progression of the previous decade [9]. This lag is mainly because these countries are still fighting economically, and they need some time to walk down the road of nanotechnology. However, it is pertinent to say that both the developed and developing world’s scientific communities agree that nanotechnology will be the next step in technological generation [10]. This will make further industrial upgrading and investment in the field of nanotechnology indispensable in the coming years.

With advances in science and technology, the scientific community adopts technologies and products that are relatively cheap, safe, and cleaner than previous technologies. Moreover, they are concerned about the financial standing of technologies, as natural resources in the world are shrinking excessively [11]. Nanotechnology thus provides a gateway to this problem. This technology is clear, cleaner, and more affordable compared to previous mass bulking and heavy machinery. Moreover, nanotechnology holds the potential to be implemented in every aspect of life. This will mainly include nanomaterial sciences, nanoelectronics, and nanomedicine, being inculcated in all dimensions of chemistry and the physical and biological world [12]. Thus, it is not wrong to predict that nanotechnology will become a compulsory field of study for future generations [13]. This review inculcates the basic applications of nanotechnology in vital industries worldwide and their implications for future industrial progress [14].

## 2. Nanotechnology Applications

### 2.1. Applications of Nanotechnology in Different Industries

After thorough and careful analyses, a wide range of industries—in which nanotechnology is producing remarkable applications—have been studied, reviewed, and selected to be made part of this review. It should be notified that multiple subcategories of industrial links may be discussed under one heading to elaborate upon the wide-scale applications of nanotechnology in different industries. A graphical abstract at the beginning of this article indicates the different industries in which nanotechnology is imparting remarkable implications, details of which are briefly discussed under different headings in the next session. 

### 2.2. Nanotechnology and Computer Industry

Nanotechnology has taken its origins from microengineering concepts in physics and material sciences [15]. Nanoscaling is not a new concept in the computer industry, as technologists and technicians have been working for a long time to design such modified forms of computer-based technologies that require minimum space for the most efficient work. Resultantly, the usage of nanotubes instead of silicon chips is being increasingly experimented upon in computer devices. Feynman and Drexler’s work has greatly inspired computer scientists to design revolutionary nanocomputers from which wide-scale advantages could be attained [13]. A few years ago, it was an unimaginable to consider laptops, mobiles, and other handy gadgets as thin as we have today, and it is impossible for even the common man to think that with the passage of time, more advanced, sophisticated, and lighter computer devices will be commonly used. Nanotechnology holds the potential to make this possible [16]. 

Energy-efficient, sustainable, and urbanized technologies have been emerging since the beginning of the 21st century. The improvement via nanotechnology in information and communication technology (ICT) is noteworthy in terms of the improvements achieved in interconnected communities, economic competitiveness, environmental stability during demographic shifts, and global development [17]. The major implications of renewable technology incorporate the roles of ICT and nanotechnology as enablers of environmental sustainability. The traditional methods of product resizing, re-functioning, and enhanced computational capabilities, due to their expensiveness and complicated manufacturing traits, have slowly been replaced by nanotechnological renovations. Novel technologies such as smart sensors logic elements, nanochips, memory storage nanodevices, optoelectronics, quantum computing, and lab-on-a-chip technologies are important in this regard [18].

Both private and public spending are increasing in the field of nanocomputing. The growth of marketing and industrialization in the biotechnology and computer industries are running in parallel, and their expected growth rates for the coming years are far higher. Researchers and technologists believe that by linking the advanced field of nanotechnology and informatics and computational industries, various problems in human society such as basic need fulfillment can be easily accomplished in line with the establishment of sustainable goals by the end of this decade [19]. The fourth industrial revolution is based upon the supporting pillars derived from hyperphysical systems including artificial intelligence, machine learning, the internet of things, robots, drones, cloud computing, fast internet technologies (5G and 6G), 3D printing, and block chain technologies [20].

Most of these technologies have a set basis in computing, nanotechnology, biotechnology, material science renovations, and satellite technologies. Nanotechnology offers useful alterations in the physiochemical, mechanical, magnetic, electrical, and optical properties of computing materials which enable innovative and newer products [21]. Thus, nanotechnology is providing a pathway for another broad-spectrum revolution in the field of automotive, aerospace, renewable energy, information technology, bioinformatics, and environmental management, all of which have root origins from nanotechnological improvements in computers. Sensors involved in software and data algorithms employ nanomaterials to induce greater sensitivity and processabilities with minimal margin-to-machine errors [22]. Nanomaterials provide better characteristics and robustness to sensor technologies which mean they are chemically inert, corrosion-resistant, and have greater tolerance profiles toward temperature and alkalinity [22].

Moreover, the use of semiconductor nanomaterials in the field of quantum computing has increased overall processing speeds with better accuracy and transmissibility. These technologies offer the creation of different components and communication protocols at the nano level, which is often called the internet of nano things [23]. This area is still in a continuous development and improvement phase with the potential for telecommunication, industrial, and medical applications. This field has taken its origin from the internet of things, which is a hyperphysical world of sensors, software, and other related technologies which allow broad-scale communication via internet operating devices [17]. The applications of these technologies range from being on the simple home scale to being on the complex industrial scale. The internet of things is mainly capable of gathering and distributing large-scale data via internet-based equipment and modern gadgets. In short, the internet of nano things is applicable to software, hardware, and network connection which could be used for data manipulation, collection, and sharing across the globe [24].

Another application of nanotechnology in the computer and information industry comes in the form of artificial intelligence, machine learning, and big data platforms which have set the basis for the fourth industrial revolution. Vast amounts of raw data are collected through interconnected robotic devices, sensors, and machines which have properties of nanomaterials [18]. After wide-scale data gathering, the next step is the amalgamation of the internet of things and the internet of people to prepare a greater analysis, understanding, and utilization of the gathered information for human benefit [4]. Such data complications can be easily understood through the use of big data in the medical industry, in which epidemiological data provide benefits for disease management [2]. Yet another example is the applications in business, where sales and retail-related data help to elucidate the target markets, sales industry, and consumer behavioral inferences for greater market consumption patterns [19].

Similarly, an important dimension of nanotechnology and computer combination comes in the form of drone and robotics technology. These technologies have a rising number of applications in maintenance, inspections, transportation, deliverability, and data inspection [25]. Drones, robots, and the internet of things are being perfectly amalgamated with the industrial sector to achieve greater goals. Drones tend to be more mobile but rely more on human control as compared to robots, which are less mobile but have larger potential for self-operation [26]. However, now, more mobile drones with better autonomous profiles are being developed to help out in the domain of manufacturing industries. These devices intensify and increase the pace of automation and precision in industries along with providing the benefits of lower costs and fewer errors [24]. The integrated fields of robotics, the internet of things, and nanotechnology are often called the internet of robotics and nano things. This field of nanorobotics is increasing the flexibility and dexterity in manufacturing processes compared to traditional robotics [25]. 

Drones, on the contrary, help to manage tasks that are otherwise difficult or dangerous to be managed by humans, such as working from a far distance or in dangerous regions. Nanosensors help to equip drones with the qualities of improved detection and sensation more precisely than previous sensor technologies [21,27]. Moreover, the over-potential of working hours, battery, and maintenance have also been improved with the operationalization of nano-based sensors in drone technology. These drones are inclusively used for various purposes such as maintaining operations, employing safety profiling, security surveys, and mapping areas [18]. However, limitations such as high speed, legal and ethical limitations, safety concerns, and greater automobility are some of the drawbacks of aerial and robotic drone technologies [26].

Three-dimensional printing is yet another important application of the nanocomputer industry, in which an integrated modus operandi works to help in production management [28]. Nanotechnology-based 3D printing offers the benefits of an autonomous, integrated, intelligent exchange network of information which enables wide-scale production benefits. These technologies have enabled a lesser need for industrial infrastructure, minimized post-processing operations, reduced waste material generation, and reduced need for human presence for overall industrial management [28,29]. Moreover, the benefits of 3D printing and similar technologies have potentially increased flexibility in terms of customized items, minimal environmental impacts, and sustainable practices with lower resource and energy consumption. The use of nano-scale and processed resins, metallic raw material, and thermoplastics along with other raw materials allow for customized properties of 3D printing technology [29].

The application of nanotechnology in computers cannot be distinguished from other industrial applications, because everything in modern industries is controlled by a systemic network in association with a network of computers and similar technologies. Thus, the fields of electronics, manufacturing, processing, and packaging, among several others, are interlinked with nanocomputer science [11,15]. Silicon tubes have had immense applications that revolutionized the industrial revolution in the 20th century; now, the industrial revolution is in yet another revolutionary phase based on nanostructures [16]. Silicon tubes have been slowly replaced with nanotubes, which are allowing a great deal of improvement and efficiency in computing technology. Similarly, lab-on-a-chip technology and memory chips are being formulated at nano scales to lessen the storage space but increase the storage volume within a small, flexible, and easily workable chip in computers for their subsequent applications in multiple other industries.

Hundreds of nanotechnology computer-related products have been marketed in the last 20 years of the nanotechnological revolution [30]. Modern industries such as textiles, automotive, civil engineering, construction, solar technologies, environmental applications, medicine, transportation agriculture, and food processing, among others are largely reaping the benefits of nano-scale computer chips and other devices. In simple terms, everything out there in nanoindustrial applications has something to do with computer-based applications in the nanoindustry [31,32,33]. Thus, all the applications discussed in this review more or less originate from nanocomputers. These applications are enabling considerable improvement and positive reports within the industrial sector. Having said that, it is hoped that computer scientists will remain engaged and will keep on collaborating with scientists in other fields to further explore the opportunities associated with nanocomputer sciences.

### 2.3. Nanotechnology and Bioprocessing Industries

Scientific and engineering rigor is being carried out to the link fields of nanotechnology with contributions to the bioprocessing industry. Researchers are interested in how the basics of nanomaterials could be used for the high-quality manufacturing of food and other biomaterials [15,34]. Pathogenic identification, food monitoring, biosensor devices, and smart packaging materials, especially those that are reusable and biodegradable, and the nanoencapsulation of active food compounds are only a few nanotechnological applications which have been the prime focus of the research community in recent years. Eventually, societal acceptability and dealing with social, cultural, and ethical concerns will allow the successful delivery of nano-based bio-processed products into the common markets for public usage [20,35].

With the increasing population worldwide, food requirements are increasing in addition to the concerns regarding the production of safe, healthy, and recurring food options. Sensors and diagnostic devices will help improve the sensitivity in food quality monitoring [36]. Moreover, the fake industrial application of food products could be easily scanned out of a system with the application of nanotechnology which could control brand protection throughout bio-processing [6]. The power usage in food production might also be controlled after a total nanotechnological application in the food industry. The decrease in power consumption would ultimately be positive for the environment. This could directly bring in the interplay of environment, food, and nanotechnology and would help to reduce environmental concerns in future [37].

One of the important implications of nanotechnology in bioprocessing industries can be accustomed to fermentation processes; these technologies are under usage for greater industrial demand and improved biomolecule production at a very low cost, unlike traditional fermentation processes [35]. The successful implementation and integration of fermentation and nanotechnology have allowed the development of biocompatible, safe, and nontoxic substances and nanostructures with wide-scale application in the field of food, bioprocessing, and winemaking industries [38]. Another important application is in the food monitoring and food supply chain management, present in various subsectors such as production, storage, distribution, and toxicity management. Nanodevices and nanomaterials are incorporated into chemical and biological sensor technologies to improve overall analytical performance with regard to parameters such as response time, sensitivity, selectivity, accuracy, and reliability [39]. The conventional methods of food monitoring are slowly being replaced with modern nano-based materials such as nanowires, nanocomposites, nanotubes, nanorods, nanosheets, and other materials that function to immobilize and label components [40]. These methods are either electrochemically or optically managed. For food monitoring, several assays are proposed and implemented with their roots in nano-based technologies; they may include molecular and diagnostic assays, immunological assays, and electrochemical and optical assays such as surface-enhanced Raman scattering and colorimetry technologies [34]. Materials ranging from heavy materials to microorganisms, pesticides, allergens, and antibiotics are easily monitored during commercial processing and bioprocessing in industries.

Additionally, nanotechnology has presented marvelous transformations in bio-composting materials. With the rising demand for biodegradable composites worldwide to reduce the environmental impact and increase the efficiency of industrial output, there is an increasing need for sustainable technologies [41]. Nanocomposites are thus being formulated with valuable mechanical properties better than conventional polymers, thus establishing their applicability in industries. The improved properties include optical, mechanical, catalytic, electrochemical, and electrical ones [42]. These biodegradable polymers are not only used in bioprocessing industries to create food products with relevant benefits but are also being deployed in the biomedical field, therapeutic industries, biotechnology base tissue engineering field, packing, sensor industries, drug delivery technology, water remediation, food industries, and cosmetics industries as well [2,24,34,43]. These nanocomposites have outstanding characteristics of biocompatibility, lower toxicities, antimicrobial activity, thermal resistance, and overall improved biodegradation properties which make them worthy of applications in products [44]. However, it is still imperative to conduct wide-scale toxicity and safety profiling for these and other nanomaterials to ensure the safety requirements, customer satisfaction, and public benefit are met [44].

Moreover, the advancement of nanotechnology has also been conferred to the development of functional food items. The exposure and integration of nanotechnology and the food industry have resulted in larger quantities of sustainable, safer, and healthier food products for human consumption, which is a growing need for the rising population worldwide [45]. The overall positive impact of nanotechnology in food processing, manufacturing, packing, pathogenic detection, monitoring, and production profiles necessitates the wide-scale application of this technology in the food industry worldwide [4,41]. Recent research has shown how the delivery of bioactive compounds and essential ingredients is and can be improved by the application of nanomaterials (nanoencapsulation) in food products [46]. These technologies improve the protection performance and sensitivity of bioactive ingredients while preventing unnecessary interaction with other constituents of foods, thus establishing clear-cut improved bioactivity and solubility profiles of nanofoods, thereby improving human health benefits. However, it should be kept in mind that the safety regards of these food should be carefully regulated with safety profiling, as they directly interact with human bodies [47].

### 2.4. Nanotechnology and Agri-Industries

Agriculture is the backbone of the economies of various nations around the globe. It is a major contributing factor to the world economy in general and plays a critical role in population maintenance by providing nutritional needs to them. As global weather patterns are changing owing to the dramatic changes caused by global warming, it is accepted that agriculture will be greatly affected [48]. Under this scenario, it is always better to take proactive measures to make agricultural practices more secure and sustainable than before. Modern technology is thus being employed worldwide. Nanotechnology has also come to play an effective role in this interplay of sustainable technologies. It plays an important role during the production, processing, storing, packaging, and transport of agricultural industrial products [49]. 

Nanotechnology has introduced certain precision farming techniques to enhance plant nutrients’ absorbance, alongside better pathogenic detection against agricultural diseases. Fertilizers are being improved by the application of nanoclays and zeolites which play effective roles in soil nutrient broths and in the restoration soil fertility [49]. Modern concepts of smart seeds and seed banks are also programmed to germinate under favorable conditions for their survival; nanopolymeric mixtures are used for coating in these scenarios [50]. Herbicides, pesticides, fungicides, and insecticides are also being revolutionized through nanotechnology applications. It has also been considered to upgrade linked fields of poultry and animal husbandry via the application of nanotechnology in treatment and disinfection practices.

### 2.5. Nanotechnology and Food Industry

**Figure 1:**
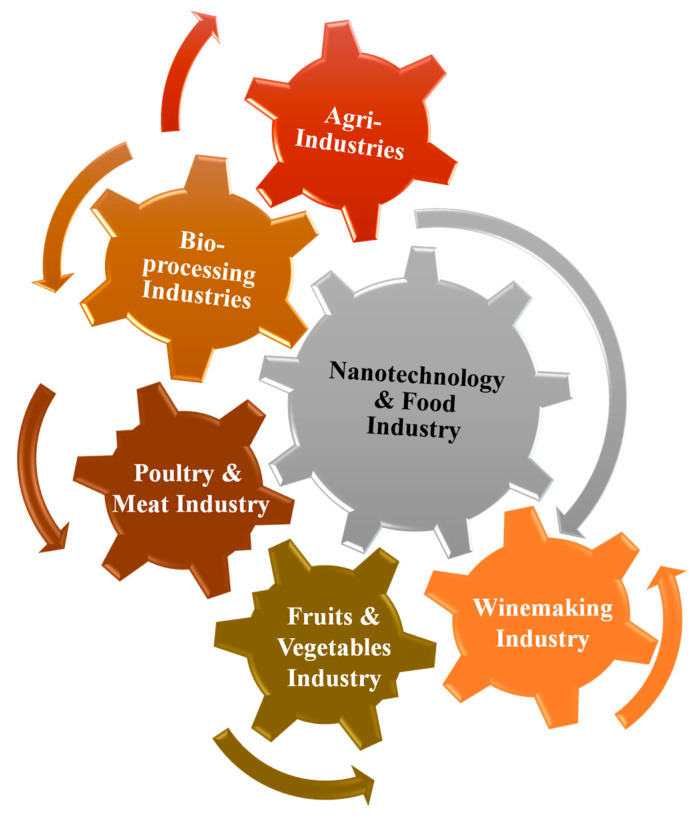
Nanotechnology applications in food and interconnected industries.

The applications of nanotechnology in the food industry are immense and include food manufacturing, packaging, safety measures, drug delivery to specific sites [51], smart diets, and other modern preservatives, as summarized in Figure 1. Nanomaterials such as polymer/clay nanocomposites are used in packing materials due to their high barrier properties against environmental impacts [52]. Similarly, nanoparticle mixtures are used as antimicrobial agents to protect stored food products against rapid microbial decay, especially in canned products. Similarly, several nanosensor and nano-assembly-based assays are used for microbial detection processes in food storage and manufacturing industries [53].

Nanoassemblies hold the potential to detect small gasses and organic and inorganic residues alongside microscopic pathogenic entities [54]. It should, however, be kept in mind that most of these nanoparticles are not directly added to food species because of the risk of toxicity that may be attached to such metallic nanoparticles. Work is being carried out to predict the toxicity attached, so that in the future, these products’ market acceptability could be increased [55]. With this, it is pertinent to say that nanotechnology is rapidly taking steps into the food industry for packing, sensing, storage, and antimicrobial applications [56]. 

Nanotechnology is also revolutionizing the dairy industry worldwide [57]. An outline of potential applications of nanotechnology in the dairy industry may include: improved processing methods, improved food contact and mixing, better yields, the increased shelf life and safety of dairy-based products, improved packaging, and antimicrobial resistance [58]. Additionally, nanocarriers are increasingly applied to transfer biologically active substances, drugs, enhanced flavors, colors, odors, and other food characteristics to dairy products [59]. 

These compounds exhibit higher delivery, solubility, and absorption properties to their targeted system. However, the problem of public acceptability due to the fear of unknown or potential side effects associated with nano-based dairy and food products needs to be addressed for the wider-scale commercialization of these products [60]. 

#### 2.5.1. Nanotechnology, Poultry and Meat Industry

The poultry industry is a big chunk of the food industry and contributes millions of dollars every year to food industries around the world. Various commercial food chains are running throughout the world, the bases of which start from healthy poultry industries. The incidence of widespread foodborne diseases that originate from poultry, milk, and meat farms is a great concern for the food industry. Nanobiotechnology is certainly playing a productive role in tackling food pathogens such as those which procreate from Salmonella and Campylobacter infections by allowing increased poultry consumption while maintaining the affordability and safety of manufactured chicken products [61]. Several nano-based tools and materials such as nano-enabled disinfectants, surface biocides, protective clothing, air and water filters, packaging materials, biosensors, and detective devices are being used to confirm the authenticity and traceability of poultry products [62]. Moreover, nano-based materials are used to reduce foodborne pathogens and spoilage organisms before the food becomes part of the supply chain [63]. 

#### 2.5.2. Nanotechnology—Fruit and Vegetable Industry

As already described, nanotechnology has made its way far ahead in the food industry. The agricultural, medicinal, and fruit and vegetable industries cannot remain unaffected under this scenario. Scientists are trying to increase the shelf life of fresh organic products to fulfill the nutritional needs of a growing population. From horticulture to food processing, packaging, and pathogenic detection technology, nanotechnology plays a vital role in the safety and production of vegetables and fruits [64]. 

Conventional technologies are now being replaced with nanotechnology due to their benefits of cost-effectiveness, satisfactory results, and overall shelf life improvement compared to past practices. Although some risks may be attached, nanotechnology has not yet reported high-grade toxicity to organic fresh green products. These technologies serve the purpose of providing safe and sufficient food sources to customers while reducing postharvest wastage, which is a major concern in developing nations [55]. Nanopackaging provides the benefits of lower humidity, oxygen passage, and optimal water vapor transmission rates. Hence, in the longer run, the shelf life of such products is increased to the desired level using nanotechnology [65].

#### 2.5.3. Nanotechnology and Winemaking Industry

The winemaking industry is a big commercial application of the food industry worldwide. The usage of nanotechnology is also expanding in this industry. Nanotechnology serves the purpose of sensing technology through employment as nanoelectronics, nanoelectrochemical, and biological, amperometric, or fluorimetric sensors. These nanomaterials help to analyze the wine components, including polyphenols, organic acids, biogenic amines, or sulfur dioxide, and ensure they are at appropriate levels during the production of wine and complete processing [66]. 

Efforts are being made to further improve sensing nanotechnology to increase the accuracy, selectivity, sensitivity, and rapid response rate for wine sampling, production, and treatment procedures [53]. Specific nanoassemblies that are used in winemaking industries include carbon nanorods, nanodots, nanotubes, and metallic nanoparticles such as gold, silver, zinc oxide, iron oxide, and other types of nanocomposites. Recent research studies have introduced the concept of electronic tongues, nanoliquid chromatography, mesoporous silica, and applications of magnetic nanoparticles in winemaking products [67]. An elaborative account of these nanomaterials is out of the scope of the present study; however, on a broader scale, it is not wrong to say that nanotechnology is successfully reaping in the field of enology.

### 2.6. Nanotechnology and Packaging Industries

The packaging industry is continuously under improvement since the issue of environmentalism has been raised around the globe. Several different concerns are linked to the packaging industry; primarily, packaging should provide food safety to deliver the best quality to the consumer end. In addition, packaging needs to be environmentally friendly to reduce the food-waste-related pollution concern and to make the industrial processes more sustainable. Trials are being carried out to reduce the burden by replacing non-biodegradable plastic packaging materials with eco-friendly organic biopolymer-based materials which are processed at the nano scale to incur the beneficial properties of nanotechnology [68]. 

The nanomanufacturing of packaging biomaterials has proven effective in food packaging industries, as nanomanufacturing not only contributes to increasing food safety and production but also tackles environmental issues [69]. Some examples of these packaging nanomaterials may include anticaking agents, nanoadditives, delivery systems for nutraceuticals, and many more. The nanocompositions of packing materials are formed by mixing nanofillers and biopolymers to enhance packaging’s functionality [70]. Nanomaterials with antimicrobial properties are preferred in these cases, and they are mixed with a polymer to prevent the contamination of the packaged material. It is important to mention here that this technology is not only limited to food packaging; instead, packaging nanotechnology is now also being introduced in certain other industries such as textile, leather, and cosmetic industries in which it is providing large benefits to those industries [64]. 

### 2.7. Nanotechnology and Construction Industry and Civil Engineering

Efficient construction is the new normal application for sustainable development. The incorporation of nanomaterials in the construction industry is increasing to further the sustainability concern [71]. Nanomaterials are added to act as binding agents in cement. These nanoparticles enhance the chemical and physical properties of strength, durability, and workability for the long-lasting potential of the construction industry. Materials such as silicon dioxide which were previously also in use are now manufactured at the nano scale [71]. These nanostructures along with polymeric additives increase the density and stability of construction suspension [72]. The aspect of sustainable development is being applied to the manufacture of modern technologies coupled with beneficial applications of nanotechnology. This concept has produced novel isolative and smart window technologies which have driven roots in nanoengineering, such as vacuum insulation panels (VIPs) and phase change materials (PCMs), which provide thermal insulation effects and thus save energy and improve indoor air quality in homes [73].

A few of the unique properties of nanomaterials in construction include light structure, strengthened structural composition, low maintenance requirements, resistant coatings, improved pipe and bridge joining materials, improved cementitious materials, extensive fire resistance, sound absorption, and insulation properties, as well as the enhanced reflectivity of glass surfaces [74]. As elaborated under the heading of civil engineering applications, concrete’s properties are the most commonly discussed and widely changing in the construction industry because of concrete’s minute structure, which can be easily converted to the nano scale [75]. More specifically, the combination of nano-SiO_2_ in cement could improve its performance in terms of compressiveness, large volumes with increased compressiveness, improved pore size distribution, and texture strength [76]. 

Moreover, some studies are also being carried out to improve the cracking properties of concrete by the application of microencapsulated healing polymers, which reduce the cracking properties of cement [77]. Moreover, some other construction materials, such as steel, are undergoing research to change their structural composites through nano-scale manufacturing. This nanoscaling improves steel’s properties such as improved corrosion resistance, increased weldability, the ease of handling for designing building materials, and construction work [78]. Additionally, coating materials have been improved by being manufactured at the nano scale. This has led to different improved coating properties such as functional improvement; anticorrosive action; high-temperature, fire, scratch, and abrasion resistance; antibacterial and antifouling self-healing capabilities; and self-assembly, among other useful applications [79].

Nanotechnology improves the compressive flexural properties of cement and reduces its porosity, making it absorb less water compared to traditional cementation preparations. This is because of the high surface-to-volume ratio of nanosized particles. Such an approach helps in reducing the amount of cement in concrete, making it more cost-effective, more strengthening, and eco-friendly, known as ‘green concrete’. Besides concrete, the revolutionary characteristics of nanotechnology are now also being adopted in other construction materials such as steel, glass, paper, wood, and multiple other engineering materials to upgrade the construction industry [80]. 

Similarly, carbon nanotubes, nanorods, and nanofibers are rapidly replacing steel constructions. These nanostructures along with nanoclay formations increase the mechanical properties and thus have paved the way for a new branch of civil engineering in terms of nanoengineering [80]. Apart from cement formulations, nanoparticles are included in repair mortars and concrete with healing properties that help in crack recovery in buildings. Furthermore, nanostructures, titanium dioxide, zinc, and other metallic oxides are being employed for the production of photocatalytic products with antipathogenic, self-cleaning, and water- and germ-repellent built-in technologies [33]. Similarly, quantum dot technologies are progressively employed for solar energy generation (a concept discussed later). These photovoltaic cells contribute to saving the maximum amount of solar energy [81].

### 2.8. Nanotechnology and Textiles Industry

The textile industry achieved glory in the 21st century with enormous outgrowth through social media platforms. Large brands have taken over the market worldwide, and millions are earned every year through textile industries. With the passing of time, nanotechnology is being slowly incorporated into the textile fiber industry owing to its unique and valuable properties. Previously, fabrics manufactured via conventional methods often curtailed the temporary effects of durability and quality [82]. However, the age of nanotechnology has allowed these fabric industries to employ nanotechnology to provide high durability, flexibility, and quality to clothes which is not lost upon laundering and wearing. The high surface-to-volume ratio of nanomaterials keeps high surface energy and thus provides better affinity to their fabrics, leading to long-term durability [82]. Moreover, a thin layering and coating of nanoparticles on the fabric make them breathable and make them smooth to the touch. This layering is carried out by processes such as printing, washing, padding, rinsing, drying, and curing to attach nanoparticles on the fabric surface. These processes are carried out to impart the properties of water repellence, soil resistance, flame resistance, hydrophobicity, wrinkle resistance, antibacterial and antistatic properties, and increased dyeability to the clothes [83]. 

The unique properties of nanomaterials in textile industries have attracted large-scale businesses for the financial benefits attached to their application. For this reason, competitors are increasing in nanotextile industry speedily, which may make the conventional textile industry sidelined in the near future [84]. Some benefits associated with nanotextile engineering and industry may include: improved cleaning surfaces, soil, wrinkle, stain, and color damage resistance, higher wettability and strike-through characteristics, malodor- and soil-removal abilities, abrasion resistance, a modified version of surface friction, and color enhancement through nanomaterials [85].

These characteristics have hugely improved the functionality and performance characteristics of textile and fiber materials [86]. Based upon the numerous advantages, nanotextile technology is increasingly being used in various inter-related fields, including in medical clothes, geotextiles, shock-resistant textiles, and fire-resistant and water-resistant textiles [87]. These textiles and fibers help overcome severe environmental conditions in special industries where high temperatures, pressure, and other conditions are adjusted for manufacturing purposes. These textiles are now increasingly called smart clothes due to renewed nanotechnological application to traditional methods [88].

The increasing demand for durable, appealing, and functionally outstanding textile products with a couple of factors of sustainability has allowed science to incorporate nanotechnology in the textile sector. These nano-based materials offer textile properties such as stain-repellent, wrinkle-free textures and fibers’ electrical conductivity alongside guaranteeing comfort and flexibility in clothing [82]. The characteristics of nanomaterials are also exhibited in the form of connected garments creation that undergo sensations to respond to external stimuli through electrical, colorant, or physiological signals. Thus, a kind of interconnection develops between the fields of photonic, electrical, textile and nanotechnologies [89]. Their interconnected applications confer the properties of high-scale performance, lasting durability, and connectivity in textile fibers. However, the concerns of nanotoxicity, the chances of the release of nanomaterials during washing, and the overall environmental impact of nanotextiles are important challenges that need to be ascertained and dealt with successfully in the coming years to ensure wide-scale acceptance and the global broad-spectrum application of nanotextiles [90].

The global market for the textile industry is constantly on the rise; with so many new brands, the competition is rising in regard to pricing, material, product outlook, and market exposure. Under this scenario, nanotechnology has contributed in terms of value addition to textiles by contributing the properties of water repellence, self-cleaning, and protection from radiation and UV light, along with safety against flames and microorganisms [82]. A whole new market of smart clothes is slowly taking our international markets along with improvements in textile machinery and economic standing. These advances have effectively established the sustainable character of the textile industry and have created grounds to meet the customer’s demand [91]. Some important examples of smart clothing originating from the nanotextile industry can be seen in products such as bulletproof jackets, fabric coatings, and advanced nanofibers. Fabric coatings and pressure pads can exhibit characteristics of invisibility and entail a silver, nickel, or gold nanoparticle-based material with inherent antimicrobial properties [92]. Such materials are effectively being utilized and introduced into the medical industry for bandages, dressings, etc. [92]. 

Similarly, woven optical fibers are already making progress in the textile and IT industry. With the incorporation of nanomaterials, optical fibers are being utilized for a range of purposes such as light transmission, sensing technologies, deformation, improved formational characteristic detection, and long-range data transmission. These optical fibers with phase-changing material properties can also be utilized for thermostability maintenance in the fiber industry. Thus, these fibers have combined applications in the computer, IT, and textile sectors [93]. In addition, the nano cellulosic material that is naturally obtained from plants confers properties of stiffness, strength, durability, and large surface area to volume ratios, which is acquired through the large number of surface hydroxyl groups embedded in nanocellulose particles [94]. Moreover, the characteristics of high resistance, lower weight, cost-effectiveness, and electrical conductivity are some additional benefits which are also linked to these nanocellulosic fibers [93]. The aforementioned technologies will allow industrialists to manufacture fabrics based on nanomaterials through a variety of chemical, physical, and biological processes. The scope of improvement in the textile properties, cost, and production methods is making the nanotextile industry a strong field of interest for future industrial investments.

### 2.9. Nanotechnology and Transport and Automobile Industry

The automotive industry is always improving its production. Nanotechnology is one such tool that could impart the automotive industry with a totally new approach to manufacturing. Automobile shaping could be improved greatly without any changes to the raw materials used. The replacement of conventional fabrication procedures with advanced nanomanufacturing is required to achieve the required outcome. Nanotechnology intends to partly renovate the automobile industry by enhancing the technical performance and reducing production costs excessively. However, there is a gap in fully harnessing the potential of nanomaterials in the automotive industry. Industrialists who were previously strict about automotive industrial principles are ready to employ novelties attached to nanotechnology to create successful applications to automobiles in the future [95]. Nanotechnology could provide assistance in manufacturing methods with an impartment of extended life properties. Cars that have been manufactured with nanotechnology applications have shown lower failure rates and enhanced self-repairing properties. Although the initial investment in the nanoautomated industry is high, the outcomes are enormous. 

The concept of sustainable transport could also be applied to the manufacturing of such nano-based technology which is CO_2_ free and imparts safe driving and quiet, clean, and wider-screen cars, which, in the future, may be called nanocars. The major interplay of nanotechnology and the automotive industry comes in the manufacturing of car parts, engines, paints, coating materials, suspensions, breaks, lubrication, and exhaust systems [32]. These properties are largely imparted via carbon nanotubes and carbon black, which renders new functionalities to automobiles. These products were previously in use, but nanoscaling and nanocoating allow for enhanced environmental, thermal, and mechanical stability to be imparted to the new generation of automobiles. In simple terms, automobiles manufactured with principal nanonovelties could result in cars with less wearing risk, better gliding potential, thinner coating lubrication requirements, and long service bodies with weight reductions [31]. These properties will ultimately reduce costs and will impart more space for improved automobile manufacturing in the future. Similarly, the development of electric cars and cars built on super capacitor technology is increasingly based on nanotechnology. The implications of nanotechnology in the form of rubber fillers, body frames made of light alloys, nanoelectronic components, nanocoatings of the interior and exterior of cars, self-repairing materials against external pressure, nanotextiles for interiors, and nanosensors are some of the nanotechnological-based implications of the automotive industry [96]. Owing to these properties, nanotechnology ventures are rapidly progressing in the automobile industry. It is expected that, soon, the automobile industry will commercialize nanotechnological perspectives on their branding strategies. 

### 2.10. Nanotechnology, Healthcare, and Medical Industry

**Figure 2:**
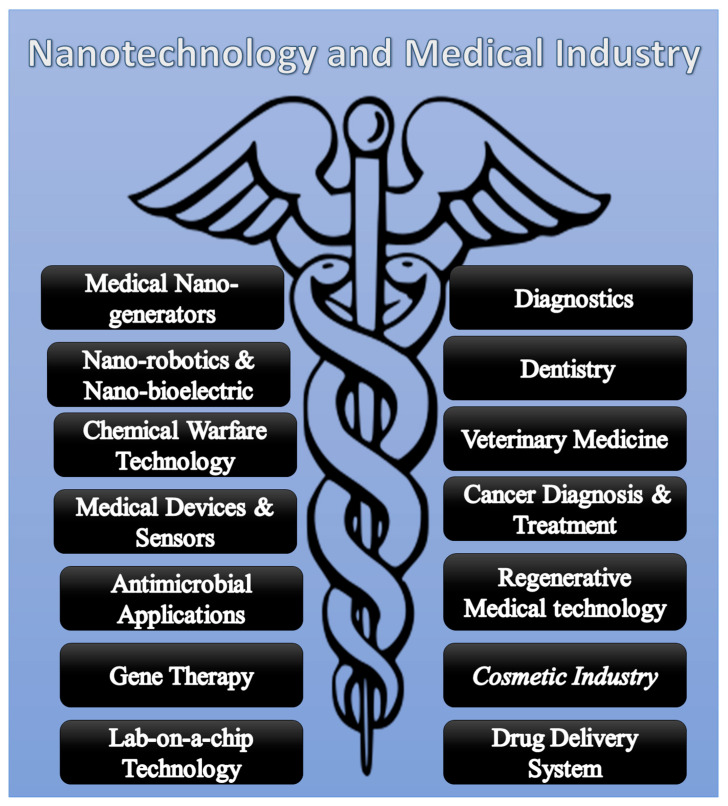
Nanotechnology applications in medical industry. Nanotechnology has a broad range of applications in various diagnostics and treatments using nanorobotics and drug delivery systems.

The genesis of nanomedicine simply cannot be ignored when we talk about the large fields of biological sciences, biotechnology, and medicine. Nanotechnology is already making its way beyond the imagination in the broader vision of nanobiotechnology. The quality of human life is continuously improved by the successful applications of nanotechnology in medicine, and resultantly, the entire new field of nanomedicine has come to the surface, which has allowed scientists to create upgraded versions of diagnostics, treatment, screening, sequencing, disease prevention, and proactive actions for healthcare [97]. These practices may also involve drug manufacturing, designing, conjugation, and efficient delivery options with advances in nano-based genomics, tissue engineering, and gene therapy. With this, it could be predicted that soon, nanomedicine will be the foremost research interest for the coming generation of biologists to study the useful impacts and risks that might be associated with them [98]. As illustrated in Figure 2, we summarized the applications of nanotechnology in different subfields of the medical industry.

In various medical procedures, scientists are exploring the potential benefits of nanotechnology. In the field of medical tools, various robotic characters have been applied which have their origins in nano-scale computers, such as diagnostic surfaces, sensor technologies, and sample purification kits [99]. Similarly, some modifications are being accepted in diagnostics with the development of devices that are capable of working, responding, and modifying within the human body with the sole purpose of early diagnosis and treatment. Regenerative medicine has led to nanomanufacturing applications in addition to cell therapy and tissue engineering [100]. Similarly, some latest technologies in the form of ‘lab-on-a-chip’, as elaborated upon earlier, are being introduced with large implications in different fields such as nanomedicine, diagnostics, dentistry, and cosmetics industries [101]. Some updated nanotechnology applications in genomics and proteomics fields have developed molecular insights into antimicrobial diseases. Moreover, medicine, programming, nanoengineering, and biotechnology are being merged to create applications such as surgical nanorobotics, nanobioelectrics, and drug delivery methods [102]. All of these together help scientists and clinicians to better understand the pathophysiology of diseases and to bring about better treatment solutions in the future. 

Specifically, the field of nanocomputers and linked devices help to control activation responses and their rates in mechanical procedures [2]. Through these mechanical devices, specific actions of medical and dental procedures are executed accurately. Moreover, programmed nanomachines and nanorobots allow medical practitioners to carry out medical procedures precisely at even sub-cellular levels [4]. In diagnostics fields, the use of such nanodevices is expanding rapidly, which allows predictions to be made about disease etiology and helps to regulate treatment options [103]. The use of in vitro diagnosis allows increased efficiency in disease apprehension. Meanwhile, in in vivo diagnoses, such devices have been made which carry out the screening of diseased states and respond to any kind of toxicities or carcinogenic or pathological irregularities that the body faces [104].

Similarly, the field of regenerative medicine is employing nanomaterials in various medical procedures such as cell therapy, tissue engineering, and gene sequencing for the greater outlook of treatment and reparation of cells, tissues, and organs. Nanoassemblies have been recorded in research for applications in powerful tissue regeneration technologies with properties of cell adhesion, migration, and cellular differentiation [102]. Additionally, nanotechnology is being applied in antimicrobial (antibacterial and antiviral) fields. The microscopic abilities of these pathogens are determined through nano-scale technologies [100]. Greek medicinal practices have long been using metals to cure pathogenic diseases, but the field of nanotechnology has presented a new method to improve such traditional medical practices; for example, nanosized silver nanomaterials are being used to cure burn wounds owing to the easy penetration of nanomaterials at the cellular level [102,105].

In the field of bioinformatics and computational biology, genomic and proteomic technologies are elucidating molecular insights into disease management [106]. The scope of targeted and personalized therapies related to pathogenic and pathophysiological diseases have greatly provided spaces for nanotechnological innovative technologies [107,108]. They also incorporate the benefits of cost-effectiveness and time saving [109]. Similarly, nanosensors and nanomicrobivores are utilized for military purposes such as the detection of airborne chemical agents which could cause serious toxic outcomes otherwise [102]. Some nanosensors also serve a purpose similar to phagocytes to clear toxic pathogens from the bloodstream without causing septic shock conditions, especially due to the inhalation of prohibited drugs and banned substances [100,105,110]. These technologies are also used for dose specifications and to neutralize overdosing incidences [110] Nano-scale molecules work as anticancer and antiviral nucleoside analogs with or without other adjuvants [21].

Another application of nanotechnology in the medical industry is in bone regeneration technology. Scientists are working on bone graft technology for bone reformation and muscular re-structuring [111,112]. Principle investigations of biomineralization, collagen mimic coatings, collagen fibers, and artificial muscles and joints are being conducted to revolutionize the field of osteology and bone tissue engineering [113,114]. Similarly, drug delivery technologies are excessively considering nanoscaling options to improve drug delivery stability and pharmacodynamic and pharmacokinetic profiles at a large scale [110]. The use of nanorobots is an important step that allows drugs to travel across the circulatory system and deliver drug entities to specifically targeted sites [99,115]. Scientists are even working on nanorobots-based wireless intracellular and intra-nucleolar nano-scale surgeries for multiple malignancies, which otherwise remain incurable [102]. These nanorobotics can work at such a minute level that they can even cut a single neuronic dendrite without causing harm to complex neuronal networks [116].

Another important application of nanotechnology in the medical field is oncology. Nanotechnology is providing a good opportunity for researchers to develop such nanoagents, fluorescent materials, molecular diagnostics kits, and specific targeted drugs that may help to diagnose and cure carcinogenesis [104]. Scientists are trying various protocols of adjoining already-available drugs with nanoparticulate conjugation to enhance drug specificity and targeting in organs [104,107,117]. Nanomedicine acts as the carrier of hundreds of specific anticancerous molecules that could be projected at tumor sites; moreover, the tumor imaging and immunotherapy approaches linked with nanomedicine are also a potential field of interest when it comes to cancer treatment management [112,117]. A focus is also being drawn toward lessening the impact of chemotherapeutic drugs by increasing their tumor-targeting efficiency and improving their pharmacokinetic and pharmacodynamic properties [112]. Similarly, heat-induced ablation treatment against cancer cells alongside gene therapy protocols is also being coupled with nanorobotics [99,118]. Anticancerous drugs may utilize the Enhanced Permeation and Retention Effect (EPR effect) by applications of nano assemblies such as liposomes, albumin nanospheres, micelles, and gold nanoparticles, which confirms effective treatment strategies against cancer [119]. Such advances in nanomedicine will bring about a more calculated, outlined, and technically programmed field of nanomedicine through association and cooperation between physicians, clinicians, researchers, and technologies.

#### 2.10.1. Nanoindustry and Dentistry 

**Figure 3:**
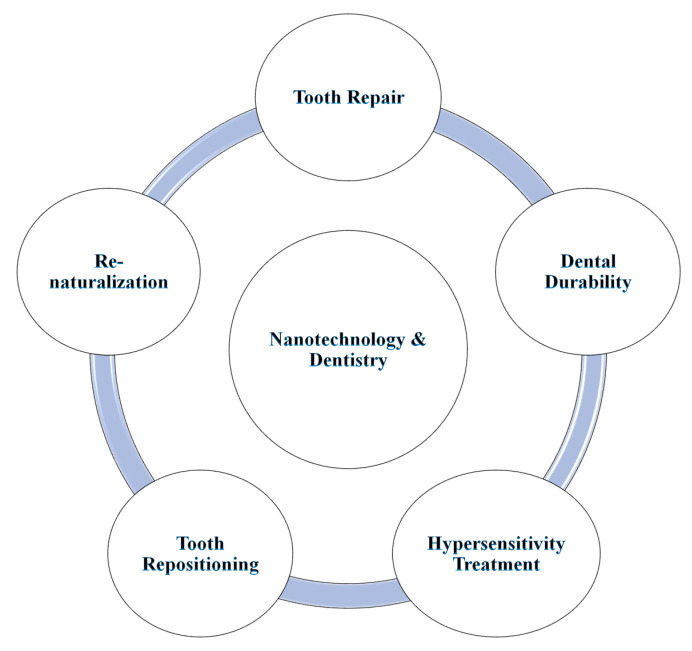
Nanotechnology applications in field of dentistry. Nanotechnology can be largely used in dentistry to repair and treat dental issues.

Nanodentistry is yet another subfield of nanomedicine that involves broad-scale applications of nanotechnology ranging from diagnosis, prevention, cure, prognosis, and treatment options for dental care [120]. Some important applications in oral nanotechnology include dentition denaturalization, hypersensitivity cure, orthodontic realignment problems, and modernized enameling options for the maintenance of oral health [2,121]. Similarly, mechanical dentifrobots work to sensitize nerve impulse traffic at the core of a tooth in real-time calculation and hence could regulate tooth tissue penetration and maintenance for normal functioning [122]. The functioning is coupled with programmed nanocomputers to execute an action from external stimuli via connection with localized internal nerve stimuli. Similarly, there are other broad-range applications of nanotechnology in tooth repair, hypersensitivity treatment, tooth repositioning, and denaturalization technologies [4,118,120,121]. Some of the applications of nanotechnology in the field of dentistry are elaborated upon in Figure 3. 

#### 2.10.2. Nanotechnology and Cosmetics Industry

The cosmetics industry, as part of the greater healthcare industry, is continuously evolving. Nanotechnology-based renovations are progressively incorporated into cosmetics industries as well. Products are designed with novel formulations, therapeutic benefits, and aesthetic output [123]. The nanocosmetics industry employs the usage of lipid nanocarrier systems, polymeric or metallic nanoparticles, nanocapsules, nanosponges, nanoemulsions, nanogels, liposomes, aquasomes, niosomes, dendrimers, and fullerenes, etc., among other such nanoparticles [101]. These nanomaterials bring about specific characteristics such as drug delivery, enhanced absorption, improved esthetic value, and enhanced shelf life. The benefits of nanotechnology are greatly captured in the improvement of skin, hair, nail, lip, and dental care products, and those associated with hygienic concerns. Changes to the skin barrier have been largely curtailed owing to the function of the nano scale of materials. The nanosize of active ingredients allows them to easily permeate skin barriers and generate the required dermal effect [124]. 

More profoundly, nanomaterials’ application is encouraged in the production of sun-protective cosmetics products such as sunblock lotions and creams. The main ingredient used is the rational combination of cinnamates (derived from carnauba wax) and titanium dioxide nanosuspensions which provide sun-protective effects in cosmetics products [125]. Similarly, nanoparticle suspensions are being applied in nanostructured lipid carriers (NLCs) for dermal and pharmaceutical applications [126]. They exhibit the properties of controlled drug-carrying and realizing properties, along with direct drug targeting, occlusion, and increased penetration and absorption to the skin surface. Moreover, these carrier nanoemulsions exhibit excellent tolerability to intense environmental and body conditions [127]. Moreover, these lipid nanocarriers have been researched and declared safe for potential cosmetic and pharmaceutical applications. However, more research is still required to assess the risk/benefit ratio of their excessive application [128].

### 2.11. Nanotechnology Industries and Environment

The environment, society, and technology are becoming excessively linked under a common slogan of sustainable development. Nanotechnology plays a key role in the 21st century to modify the technical and experimental outlook of various industries. Environmental applications cannot stand still against revolutionary applications of nanotechnology. Since the environment has much to do with the physical and chemical world around a living being, the nano scale of products greatly changes and affects environmental sustainability [129]. The subsequent introduction of nanomaterials in chemistry, physics, biotechnology, computer science, and space, food, and chemical industries, in general, directly impacts environmental sciences.

With regard to environmental applications, the remarkable research and applications of nanotechnology are increasing in the processing of raw materials, product manufacturing, contaminate treatment, soil and wastewater treatment, energy storage, and hazardous waste management [130]. In developed nations, it is now widely suggested that nanotechnology could play an effective role in tackling environmental issues. In fact, the application of nanotechnology could be implemented for water and cell cleaning technologies, drinking safety measures, and the detoxification of contaminants and pollutants from the environment such as heavy metals, organochlorine pesticides, and solvents, etc., which may involve reprocessing although nanofiltration. Moreover, the efficiency and durability of materials can be increased with mechanical stress and weathering phenomena. Similarly, the use of nanocage-based emulsions is being used for optical imaging techniques [131]. 

In short, the literature provides immense relevance to how nanotechnology is proving itself through groundbreaking innovative technologies in environmental sciences. The focus, for now, is kept on remediation technologies with prime attention on water treatment, since water scarcity is being faced worldwide and is becoming critical with time. There is a need for the scientific community to actively conduct research on comprehending the properties of nanomaterials for their high surface area, related chemical properties, high mobility, and unique mechanical and magnetic properties which could be used for to achieve a sustainable environment [132]. 

### 2.12. Nanotechnology—Oil and Gas Industry

The oil and gas industry makes up a big part of the fossil industry, which is slowly depleting with the rising consumption. Although nanotechnology has been successfully applied to the fields of construction, medicine, and computer science, its application in the oil and gas industry is still limited, especially in exploration and production technologies [133]. The major issue in this industry is to improve oil recovery and the further exploitation of alternative energy sources. This is because the cost of oil production and further purification is immense compared to crude oil prices. Nanotechnologists believe that they could overcome the technological barriers to developing such nanomaterials that would help in curtailing these problems.

Governments are putting millions of dollars into the exploration, drilling, production, refining, wastewater treatment, and transport of crude oil and gas. Nanotechnology can provide assistance in the precise measurement of reservoir conditions. Similarly, nanofluids have been proven to exhibit better performance in oil production industries. Nanocatalyses enhance the separation processing of oil, water, and gases, thus bringing an efficient impurity removal process to the oil and gas industry. Nanofabrication and nanomembrane technologies are excessively being utilized for the separation and purification of fossil materials [134]. Finally, functional and modified nanomaterials can produce smart, cost-effective, and durable equipment for the processing and manufacturing of oil and gas. In short, there is immense ground for the improvement of the fossil fuel industry if nanotechnology could be correctly directed in this industry [135]. 

### 2.13. Nanotechnology and Renewable Energy (Solar) Industry

Renewable energy sources are the solutions to many environmental problems in today’s world. This makes the renewable energy industry a major part of the environmental industry. Subsequently, nanotechnology needs to be considered in the energy affairs of the world. Nanotechnologies are increasingly applied in solar, hydrogen, biomass, geothermal, and tidal wave energy production. Although, scientists are convinced that much more needs to be discovered before enhancing the benefits of coupled nanotechnology and renewable energy [136].

Nanotechnology has procured its application way down the road of renewable energy sources. Solar collectors have been specifically given much importance since their usage is encouraged throughout the world, and with events of intense solar radiation, the production and dependence of solar energy will be helpful for fulfilling future energy needs. Research data are available regarding the theoretical, numerical, and experimental approaches adopted for upgrading solar collectors with the employment of nanotechnologies [137]. 

These applications include the nanoengineering of flat solar plates, direct absorption plates, parabolic troughs, and wavy plates and heat pipes. In most of these instruments and solar collection devices, the use of nanofluids is becoming common and plays a crucial role in increasing the working efficiency of these devices. A gap, however, exists concerning the usage of nanomaterials in the useful manufacturing design of solar panels and their associated possible efficiencies which could be brought to the solar panel industry. Moreover, work needs to be done regarding the cost-effectiveness and efficiency analyses of traditional and nanotechnology-based solar devices so that appropriate measures could be adopted for the future generation of nanosolar collectors [138].

### 2.14. Nanotechnology and Wood Industry

The wood industry is one of the main economic drivers in various countries where forest growth is immense and heavy industrial setups rely on manufacturing and selling wood-based products [139]. However, the rising environmental concerns against deforestation are a major cause for researchers to think about a method for the sustainable usage of wood products. Hence, nanotechnology has set its foot in the wood industry in various applications such as the production of biodegradable materials in the paper and pulp industry, timber and furniture industry, wood preservatives, wood composites, and applications in lignocellulosic-based materials [140]. Resultantly, new products are introduced into the market with enhanced performance (stronger yet lighter products), increased economic potential, and reduced environmental impact.

One method of nano-based application in the wood industry is the derivation of nanomaterials directly from the forest, which is now called nanocellulose material, known broadly for its sustainable characteristics [141]. This factor has pushed the wood industry to convert cellulosic material to nanocellulose with increased strength, low weight, and increased electromagnetic response along with a larger surface area [142]. These characteristics are then further used as reinforcing agents in different subcategories of wood-based industries, including substrate, stabilizer, electronics, batteries, sensor technologies, food, medicine, and cosmetics industries [143]. Moreover, functional characteristics such as the durability, UV absorption, fire resistance, and decreased water absorption of wood-based biodegradable products are also being improved with the application of nanomaterials such as nanozinc oxide or nanotitanium oxide [144]. Similarly, wood biodegradable properties are reduced through the application of nanoencapsulated preservatives to improve the impregnation of wood with the increasing penetration of applied chemicals and a reduced leaching effect.

Cellulosic nanomaterials exhibit nanofibrillar structures which can be made multifunctional for application in construction, furniture, food, pharmaceuticals, and other wood-based industries [145]. Research is emerging in which promising results are predicted in different industries in which nanofibers, nanofillers, nanoemulsions, nanocomposites, and nano-scaled chemical materials are used to increase the potential advantages of manufactured wood products [146]. The outstanding properties of nanocellulusice materials have largely curtailed the environmental concerns in the wood industry in the form of their potential renewable characteristics, self-assembling properties, and well-defined architecture. However, there are a few challenges related to such industries, such as cost/benefit analyses, a lack of compatibility and acceptability from the public owing to a lack of proper commercialization, and a persistent knowledge gap in some places [145]. Therefore, more effort is required to increase the applications and acceptability of nano-based wood products in the market worldwide.

### 2.15. Nanotechnology and Chemical Industries

Nanotechnology can be easily applied to various chemical compositions such as polymeric substances; this application can bring about structural and functional changes in those chemical materials and can address various industrial applications including medicine, physics, electronics, chemical, and material industries, among others [76,132,138]. One such industrial application is in electricity production, in which different nanomaterials driven from silver, golden, and organic sources could be utilized to make the overall production process cheaper and effective [147]. Another effective application is in the coatings and textile industry, which has already been discussed briefly. In these industries, enzymatic catalysis in combination with nanotechnology accelerates reaction times, saving money and bringing about high-quality final products. Similarly, the water cleaning industry can utilize the benefits of nanomaterials in the form of silver and magnetic nanoparticles to create strong forces of attraction that easily separate heavy material from untreated water [148]. Similarly, there is a wide range of chemicals that can be potentially upgraded, although the nano scale for application in biomedical industries is discussed under the heading of nanotechnology and medicine.

Another major application of nanotechnology in the chemical industry includes the surfactant industry, which is used for cleaning paper, inks, agrochemicals, drugs, pharmaceuticals, and some food products [149]. The traditional surfactant application was of great environmental and health concern, but with the newer and improved manufacturing and nanoscaling of surfactants, environmentally friendly applications have been made possible. These newer types may include biosurfactants obtained via the process of fermentation and bio-based surfactants produced through organic manufacturing. More research is required to establish the risks and side effects of these nanochemical agents [3].

## 3. Closing Remarks

Nanotechnology, within a short period, has taken over all disciplinary fields of science, whether it is physics, biology, or chemistry. Now, it is predicted to enormously impact manufacturing technology owing to the evidential and proven benefits of micro scaling. Every field of industry, such as computing, information technology, engineering, medicine, agriculture, and food, among others, is now originating an entire new field in association with nanotechnology. These industries are widely known as nanocomputer, nanoengineering, nanoinformatics, nanobiotechnology, nanomedicine, nanoagriculture, and nanofood industries. The most brilliant discoveries are being made in nanomedicine, while the most cost-effective and vibrant technologies are being introduced in materials and mechanical sciences.

The very purpose of nanotechnology, in layman’s terms, is to ease out the manufacturing process and improve the quality of end products and processes. In this regard, it is easy and predictable that it is not difficult for nanotechnology to slowly take out most of the manufacturing process for industrial improvement. With every coming year, more high-tech and more effective-looking nanotechnologies are being introduced. This is smoothing out the basis of a whole new era of nanomindustries. However, the constructive need is to expand the research basis of nanoapplications to entail the rigorous possible pros of this technology and simultaneously figure out a method to deal with the cons of the said technology.

The miniaturization of computer devices has continued for many years and is now being processed at the nanometer scale. However, a gap remains to explore further options for the nanoscaling of computers and complex electronic devices, including computer processors. Moreover, there is an immense need to enable the controlled production and usage of such nanotechnologies in the real world, because if not, they could threaten the world of technology. Scientists should keep on working on producing nanoelectronic devices with more power and energy efficiency. This is important in order to extract the maximum benefits from the hands of nanotechnology and computer sciences [5]. 

Under the influence of nanotechnology, food bioprocessing is showing improvement, as proven by several scientific types of research and industrial applications in food chain and agricultural fields. Moreover, the aspect of sustainability is being introduced to convert the environment, food chains, processing industries, and production methods to save some resources for future generations. The usage of precision farming technologies based upon nanoengineering, modern nano-scale fertilizers, and pesticides are of great importance in this regard. Moreover, a combined nanotechnological aspect is also being successfully applied to the food industry, affecting every dimension of packing, sensing, storage, manufacturing, and antimicrobial applications. It is pertinent to say that although the applications of nanotechnology in the food, agriculture, winemaking, poultry, and associated packaging industries are immense, the need is to accurately conduct the risk assessment and potential toxicity of nanomaterials to avoid any damage to the commercial food chains and animal husbandry practices [63]. 

The exposure of the nano-based building industry is immense for civil and mechanical engineers; now, we need to use these technologies to actually bring about changes in those countries in which the population is immense, construction material is depleting, and environmental sustainability problems are hovering upon the state. By carefully assessing the sustainability potential of these nanomaterials, their environmental, hazardous, and health risks could be controlled, and they could likely be removed from the construction and automobile industry all over the world with sincere scientific and technical rigor [150]. It is expected that soon, the construction and automobile industry will commercialize the nanotechnological perspectives alongside sustainability features in their branding strategies. These nano-scale materials could allow the lifecycle management of automotive and construction industries with the provision of sustainable, safe, comfortable, cost-effective, and more eco-friendly automobiles [32]. The need is to explore the unacknowledged and untapped potential of nanotechnology applications in these industry industries.

Similarly, nanotechnology-based applications in consumer products such as textile and esthetics industries are immense and impressive. Professional development involves the application of nanotechnology-based UV-protective coatings in clothes which are of utmost need with climatic changes [73]. The application of nanotechnology overcomes the limitations of conventional production methods and makes the process more suitable and green-technology-based. These properties have allowed the textile companies to effectively apply nanotechnology for the manufacture of better products [90]. With greater consumer acceptability and market demand, millions are spent in the cosmetic industry to enable the further usage of nanotechnology. Researchers are hopeful that nanotechnology would be used to further upgrade the cosmetics industry in the near future [123]. 

Furthermore, the breakthrough applications of nanomedicine are not hidden from the scientific community. If nanomedicine is accepted worldwide in the coming years, then the hope is that the domain of diagnosis and treatment will become more customized, personalized, and genetically targeted for individual patients. Treatment options will ultimately become excessive in number and more successful in accomplishment. However, these assumptions will stay a dream if the research remains limited to scientific understanding. 

The real outcome will be the application of this research into the experimental domain and clinical practices to make them more productive and beneficial for the medical industry. For this cause, a combined effort of technical ability, professional skills, research, experimentation, and the cooperation of clinicians, physicians, researchers, and technology is imperative. However, despite all functional beneficial characteristics, work needs to be done and more exploration is required to learn more about nanotechnology and its potential in different industries, especially nanomedicine, and to take into account and curtail the risks and harms attached to the said domain of science.

Additionally, climatic conditions, as mentioned before, along with fossil fuel depletion, have pushed scientists to realize a low-energy-consuming and more productive technological renovation in the form of nanoengineered materials [48]. Now, they are employing nanomaterials to save energy and harvest the maximum remaining natural resources. There is immense ground for the improvement of the fossil fuel industry if nanotechnology could be correctly directed in this industry [135]. The beneficial applications within the solar industry, gas and oil industry, and conversion fields require comparative cost-effectiveness and efficiency analyses of traditional and nano-based technologies so that appropriate measures could be adopted for the future generation of nano-based products in said industries [138].

As every new technology is used in industries, linked social, ethical, environmental, and human safety issues arise to halt the pace of progress. These issues need to be addressed and analyzed along with improving nanotechnology so that this technology easily incorporates into different industries without creating social, moral, and ethical concerns. Wide-scale collaboration is needed among technologists, engineers, biologists, and industrials for a prospective future associated with the wide-scale application of nanotechnology in diversified fields. 

## 4. Conclusions

Highly cost-effective and vibrant nanotechnologies are being introduced in materials and mechanical sciences. A comprehensive overview of such technologies has been covered in this study. This review will help researchers and professionals from different fields to delve deeper into the applications of nanotechnology in their particular areas of interest. Indeed, the applications of nanotechnology are immense, yet the risks attached to unlimited applications remain unclear and unpronounced. Thus, more work needs to be linked and carefully ascertained so that further solutions can be determined in the realm of nanotoxicology. Moreover, it is recommended that researchers, technicians, and industrialists should cooperate at the field and educational level to explore options and usefully exploit nanotechnology in field experiments. Additionally, more developments should be made and carefully assessed at the nano scale for a future world, so that we are aware of this massive technology. The magnificent applications of nanotechnology in the industrial world makes one think that soon, the offerings of nanotechnology will be incorporated into every possible industry. However, there is a need to take precautionary measures to be aware of and educate ourselves about the environmental and pollution concerns alongside health-related harms to living things that may arise due to the deviant use of nanotechnology. This is important because the aspect of sustainability is being increasingly considered throughout the world. So, by coupling the aspect of sustainability with nanotechnology, a prosperous future of nanotechnology can be guaranteed. 

## Data Availability

Not applicable.
